# Acylated *mono*-, *bis*- and *tris*- Cinchona-Based Amines Containing Ferrocene or Organic Residues: Synthesis, Structure and *in Vitro* Antitumor Activity on Selected Human Cancer Cell Lines

**DOI:** 10.3390/molecules17032316

**Published:** 2012-02-24

**Authors:** Benedek Imre Károlyi, Szilvia Bősze, Erika Orbán, Pál Sohár, László Drahos, Emese Gál, Antal Csámpai

**Affiliations:** 1 Institute of Chemistry, Eötvös Loránd University, P. O. B. 32, H-1518 Budapest-112, Hungary; Email: karolbim@caesar.elte.hu (B.I.K.); sohar@chem.elte.hu (P.S.); 2 Research Group of Peptide Chemistry, Hungarian Academy of Sciences, Eötvös Loránd Universiy, P. O. B. 32, H-1518 Budapest-112, Hungary; Email: bosze@chem.elte.hu (S.B.); eorban2@chem.elte.hu (E.O.); 3 Chemres Institute of Structural Chemistry Chemical Research Center, Hungarian Academy of Sciences, H-1025 Budapest, Pusztaszeri str. 59-67, Hungary; Email: drahos@chemres.hu; 4 Faculty of Chemistry and Chemical Engineering, Babes-Bolyai University, Arany János str. 11, 400028 Cluj-Napoca, Romania; Email: gal.szabo.emese@gmail.com

**Keywords:** quinine, ferrocene, axial, symmetry, anticancer, activity, *in vitro* assay

## Abstract

A series of novel functionalized *mono*-, *bis*- and *tris-*(*S*)-{[(2*S*,4*R*,8*R*)-8-ethyl-quinuclidin-2-yl](6-methoxyquinolin-4-yl)}methanamines including ferrocene-containing derivatives was obtained by the reaction of the precursor amine with a variety of acylation agents. Their *in vitro* antitumor activity was investigated against human leukemia (HL-60), human neuroblastoma (SH-SY5Y), human hepatoma (HepG2) and human breast cancer (MCF-7) cells by the 3-(4,5-dimethylthiazol-2-yl)-2,5-diphenyltetrazolium bromide (MTT)-assay and the 50% inhibitory concentration (IC_50_) values were determined. Our data indicate that the precursor amine has no antitumor activity *in vitro*, but the *bis*-methanamines with ureido-, thioureido and amide-type linkers display attractive *in vitro* cytotoxicity and cytostatic effects on HL-60, HepG2, MCF-7 and SH-SY5Y cells. Besides ^1^H- and ^13^C-NMR methods the structures of the new model compounds were also studied by DFT calculations.

## 1. Introduction

Chemotherapy is one of the most important methods in fighting cancer and several members of modified natural alkaloids serve as deserving drugs against tumors. Well-known representatives of vinca alkaloids such as vinblastine, camptothecine, staurosporine and ellipticin [1,2,3,4] are typical examples. Cinchona alkaloids have been proved to be efficient antimalarial [5] and antibacterial drug candidates [6]. It is well-documented that the application of quinine derivatives in the field of cancer detection [7,8] and in chemotherapy [9,10,11,12,13,14] goes far back to the past. Since ferrocene-based molecules as anti-tumor agents are also promising matherials [15,16,17,18,19,20,21] with a wide range of biological activities [22] first we envisaged the synthesis of novel ferrocene-based *mono*- and *bis*-quinines containing amide, urea, thiourea and acylthiourea linkers providing hydrophilic character along with different hydrogen bond profile for the models subjected to *in vitro* assays. This choice of functional groups can also be reasoned by the following facts: (i) a number of aromatic urea derivatives play important role as anticancer agents [23]; (ii) similarly, urea-based prodrugs have been reported as candidates for melanocyte-directed enzyme therapy [24]; (iii) thiourea based molecules have been proved to be effective agents in the treatment of human promyelocytic leukemia [25]; (iv) the antiproliferative activity [26] and citotoxicity [27] of some acyl-thiourea derivatives are worth to be noted and a few patents have also been published in this field [28,29].

The pronounced efficiency of several drugs with C_2_-symmetry [30,31,32] and *bis*-quinolines [33,34,35] encouraged us to construct three ferrocene derivatives containing two quinine units with C_2_-symmetry and a reference benzene 1,3,5-tricarboxamide incorporating three quinine units with C_3_-symmetry. Two further purely organic models with one- and two quinine moieties, respectively, were also prepared as additional references.

## 2. Results and Discussion

### 2.1. Synthesis of the Model Compounds

For each synthesis reported in this contribution (*S*)-{[(2*S*,4*R*,8*R*)-8-ethylquinuclidin-2-yl](6-methoxyquinolin-4-yl)}methanamine (**1**) [36,37] was used as common precursor serving as source of quinine moiety. In the presence of dimethylaminopyridine (DMAP) the treatment of **1** with the corresponding acylating agent (fluorocarbonylferrocene, 1,1'-*bis*-fluorocarbonylferrocene, 1,3,5-* tris*-chlorocarbonylbenzene) in DCM afforded amides **2**, **3** and **8**. In dry THF the additions of **1** on heterocumulene-type reactants (1,1'-*bis*-isocyanatoferrocene, benzoylisothiocyanate and ferrocene-1,1'-*bis*-carbonylisothiocyanate) resulted in the formation of urea- and *N*-acylisothiocyanate derivatives **4** and **5**, **6**. The purely organic thiourea model **7** of C_2_-symmetry was obtained by using thiocarbonyl-diimidazole (TCDI) as reagent. Unstable heterocumulenes 1,1'-bis-isothiocyanato-carbonylferrocene and benzoylisothiocyanate obtained by the reactions of potassium isothiocyanate in acetone at 25 °C with 1,1'-*bis*-chlorocarbonylferrocene and benzoylchloride, respectively, were used without purification. The low yields of **4**–**6** (2%–10%) may be ascribed to a variety of competitive transformations including acylation- and bridging reactions along with uncontrolled polymerization processes. Their purification required repeated column chromatography and recrystallization until the ^1^H-NMR spectra displayed no major impurities.

**Scheme 1:**
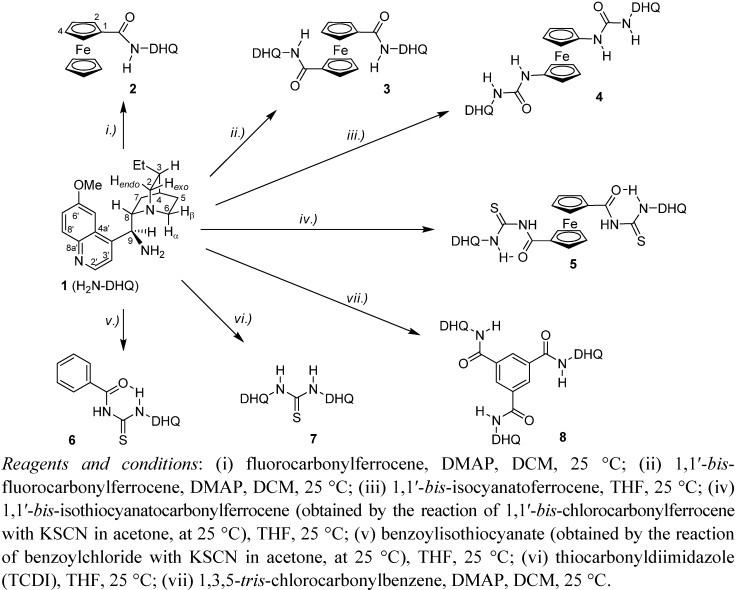
Synthesis of ferrocene-based- and purely organic quinine derivatives.

### 2.2. Theoretical Calculations

Since the attempts to grow crystals suitable for X-ray analysis have failed so far, the preferred conformations of the new compounds with potential relevance for receptor binding properties were examined by routine DFT calculations [38]. The geometry optimization of the *bis*- and *tris*-cinchona derivatives (**3**–**5**, **7** and **8**) was carried out using the appropriate symmetry constraint (C_n_ n = 2,3). It was found that in each model compound the N-1 atom is situated in the proximity of the hydrogen atom of the NH group directly attached to the cinchona skeleton (calculated distances: 2.1–2.3 Å) to form a five membered chelate ring representing a hydrophilic segment of the molecule. As evidenced by its downfield-shifted ^1^H-NMR signal, this NH group is also incorporated in an additional six-membered chelate ring in the acylthiourea derivatives **5** and **6** considerably decreasing the flexibility of these molecules. In the optimized structures of **2**–**5** the Cp-rings are in the eclipsed position relative to each other and the attached functional groups (amide, urea and acylthiourea) are practically coplanar with them (see Supporting Data for the atomic coordinates).

### 2.3. Structure Determination

The supposed structures of the new compounds investigated **2**–**8** are consistent with their spectral parameters, so only the following remarks are necessary: the C_2_- and C_3_-symmetric structures of **3**–**5**, **7** and **8**, respectively, are confirmed by the identical spectral data of the two or three chincona residues incorporated in these compounds. In acylthioureas **5** and **6** the presence of the chelate rings gains support from the significantly downfield-shifted ^1^H-NMR signal of the NH group directly bonded to C-9 atom. The relative configuration of the cinchona unit in each compound investigated was evidenced by DNOE measurements revealing *endo* position of H-9 in the proximity of H-5_α_- and H-7_α_, respectively. Accordingly, NOE’s were detected between H-6_α_ and the proton of the NH group attached to C-9 atom. Their proximity is also reflected from the significant downfield shift of the H-6_α_ signal relative to that of its germinal partner, H-6_β_ (Δδ = 0.6–0.7 ppm). The relative configuration of C-8 centre gains support from the NOE interactions measured between H-2*_endo_*- and H-8 atoms. On irradiation of the protons on the ethyl group significant enhancements of the intensity of H-7_β_ and H-8 signals were observed indicating the relative configuration of the C-3 atom.

### 2.4. *In Vitro* Activity of the Compounds on Human Tumor Cell Cultures

We have determined the cytotoxic and cytostatic activity of the compounds *in vitro* on four human tumor cell lines: HL-60 leukemia, HepG2 hepatoma, MCF-7 breast adenocarcinoma and SH-SY5Y neuroblastoma cell cultures and expressed them as IC_50_ values. Therefore cells were treated with the compounds at 10^−4^ to 10^2^μM concentration range and the viability of the cells was determined by MTT-assay.

The data summarized in Table 1 show that the precursor amine **1** has no antitumor activity *in vitro* on the tested human cancer cell cultures. Among the investigated ferrocene derivatives **2–5** the diamide **3** proved to be the most active on each type of tumor cells (the IC_50_ values of its cytotoxic- and cytostatic effects fall into the ranges between 0.72–1.70 µM and 0.40–1.00 µM, respectively). It is worth to emphasize that the presence of an additional quinine amide moiety in **3** induces a dramatic enhancement in the *in vitro* antitumor activity compared to that of the analogue *mono*-amide **2**. The outstanding efficiency of **3** can probably be attributed to a cooperation of the two functionalities adopting optimal conformation by practically unrestricted rotation of the two Cp rings.

Significant differences are discernible between the activities of ferrocene-based *bis*-urea **4** and *bis*-acylthiourea **5**. While **4** shows considerable activities on each investigated cell line, compound **5** has only selective cytotoxic and cytostatic effect on the HL-60 cells. The spectacularly decreased activity of **5** may be associated with the intramolecular hydrogen bonds and the increased rigidity of the two acylthiourea units stabilized by their chelate structure.

Among the purely organic models thiourea **7** of C_2_-symmetry exhibited significant cytotoxic and cytostatic effects against each tested cancer cell line, especially on HL-60 and SH-SY5Y cultures (cytotoxic effect: IC_50_ = 1.80 and 0.84 µM, cytostatic activity: 10.20 and 4.20 µM, respectively). Acylthiourea **6** and *tris*-amide **8** with C_3_-symmetry also displayed remarkable activities (with higher IC_50_ values: Between 17.60 and 32.20 µM; 2.30 and 8.90 µM, respectively) without *in vitro* cytotoxic effect on MCF-7 cells. On the other hand, these molecules were slightly cytostatic on the same cell lines after overnight incubation (Table 1).

**Table 1:** *In vitro* cytotoxicity and cytostatic activity of the cinchona derivatives on human tumor cell cultures.

	**Cell line**			
	HepG2	SH-SY5Y	HL-60	MCF-7
**Compd.**	**Cytotoxicity (IC_50_^a^ in µM)**			
**1**	>100	>100	>100	> 100
**2**	33.10 ± 3.04	29.80 ± 4.24	37.70 ± 3.67	25.32 ± 4.60
**3**	0.72 ± 0.01	0.78 ± 0.02	1.70 ± 0.05	0.75 ± 0.02
**4**	4.24 ± 1.12	0.82 ± 0.54	0.86 ± 0.02	21.70 ± 3.23
**5**	>100	>100	6.70 ± 0.02	>100
**6**	17.60 ± 0.25	21.20 ± 3.24	32.20 ± 4.67	>100
**7**	3.34 ± 1.02	0.84 ± 0.02	1.80 ± 0.56	5.34 ± 1.78
**8**	8.90 ± 0.23	1.50 ± 0.02	2.30 ± 0.05	>100
	**Cytostatic effect (IC_50_ in µM)**			
**1**	>100	>100	>100	> 100
**2**	65.00 ± 6.70	80.70 ± 5.78	41.90 ± 1.45	56.00 ± 4.56
**3**	0.40 ± 0.17	0.99 ± 0.10	0.76 ± 0.01	1.00 ± 0.34
**4**	3.40 ± 0.12	1.30 ± 0.54	0.94 ± 0.02	5.10 ± 0.67
**5**	>100	>100	6.50 ± 3.56	21.80 ± 3.18
**6**	65.60 ± 3.40	82.90 ± 6.78	>100	82.90 ± 7.98
**7**	4.60 ± 0.02	4.20 ± 2.30	10.20 ± 1.65	3.89 ± 1.18
**8**	19.60 ± 2.12	17.20 ± 3.45	4.50 ± 0.01	2.36 ± 0.01

^a^ The 50% inhibitory concentration (IC_50_) values were determined from the dose-response curves. The curves were defined using Microcal^TM^ Origin1 (version 7.5) software.

## 3. Experimental

### 3.1. General

All chemicals were obtained from commercially available sources (Sigma-Aldrich) and–except for THF–used without further purification. THF was purified by distillation from LiAlH_4_ under inert atmosphere. For the *in vitro* assays 3-(4,5-dimethylthiazol-2-yl)-2,5-diphenyltetrazolium bromide [MTT], RPMI-1640 medium, DMEM medium, fetal calf serum [FCS] and nonessential amino acids were also obtained from Sigma-Aldrich. Melting points (uncorrected) were determined with a Boethius microstage. Merck Kieselgel (230–400 mesh, 60 Ǻ) and alumina (Brockmann I grade, approx. 150 mesh, 58 Ǻ, activated neutral). The reactions were monitored using standard TLC technique and were stopped when no more starting compound was detected.

HL-60 human leukemia cells (ATCC: CCL-240) and the adherent HepG2 human hepatoma cells (ATCC: HB-8065) were cultured in RPMI-1640 medium supplemented with 10% FCS (fetal calf serum, Sigma Ltd.), 2 mM l-glutamine, and 160 μg/mL gentamycin. The adherent MCF-7 human breast adenocarcinoma cells (ATCC: HTB-22) and the adherent SH-SY5Y human neuroblastoma cells were maintained in DMEM medium containing 10% FCS, L-glutamine (2 mM), gentamycin (160 μg/mL), 1 mM pyruvate and 1% nonessential amino acids. Cell cultures were maintained at 37 °C in a humidified atmosphere with 5% CO_2_.

The IR spectra were run in KBr disks on a Bruker IFS-55 FT-spectrometer controlled by Opus 3.0 software. Optical rotations were measured with a Zeiss Polamat A polarimeter. The ^1^H- and ^13^C-NMR spectra were recorded in CDCl_3_ or DMSO-d_6_ solution in 5 mm tubes at RT, on a Bruker DRX-500 spectrometer at 500.13 (^1^H) and 125.76 (^13^C) MHz, with the deuterium signal of the solvent as the lock and TMS as internal standard. DEPT spectra were run in a standard manner, using only a *Θ* = 135° pulse to separate the CH/CH_3_ and CH_2_ lines phased “up” and “down”, respectively. The 2D-COSY, HMQC and HMBC spectra were obtained by using the standard Bruker pulse programs. The exact mass measurements were performed using a Q-TOF Premier mass spectrometer (Waters Corporation, 34 Maple St, Milford, MA, USA) in positive electrospray mode.

The precursor [(*S*)-((2*S*,4*R*,8*R*)-8-ethylquinuclidin-2-yl](6-methoxyquinolin-4-yl)methanamine (**1**) was prepared according to the method described by Brunner *et al.* [36] and is simply referred to as “amine” in each procedure described below.

### 3.2.Synthesis of the Novel Quinine Derivatives

#### 3.2.1. N*-{(*S*)-[(2*S*,4*R*,8*R*)-8-Ethylquinuclidin-2-yl](6-methoxyquinolin-4-yl)methyl)}ferrocene-carboxamide* (**2**)

The amine (0.70 g, 2.2 mmol), fluorocarbonylferrocene (0.50 g, 2.2 mmol; prepared from ferrocene carboxylic acid according to the method reported by Galow *et al.* [39]) and dimethylaminopyridine (DMAP; 0.26 g, 2.2 mmol) were dissolved in DCM (10 mL). the solution was stirred at RT under argon for 45 min and evaporated to dryness. The residue was subjected to column chromatography on silica [eluent: DCM/MeOH (30:1)] followed by recrystallization from dry ether (using 25 mL for 100 mg substance) to obtain the product as light yellow powder (592 mg, 51%). mp. 184–186 °C; [α]_D_^26^: −46.4° (EtOH *c *= 0.23 g/100 mL); IR (cm^−1^): 3313, 1635, 1530, 1512, 1242, 1174, 1029, 581, 489; ^1^H-NMR (DMSO-d_6_): 8.74 (d, 1H, *J* = 4.5 Hz, H-2'); 7.93 (d, 1H, *J* = 9.2 Hz, H-8'); 7.90 (br s, 1H, NH); 7.88 (d, *J *= 7.25 Hz, 1H, H-5'); 7.60 (d, *J *= 4.5 Hz, H-3'); 7.40 (d, 1H, *J* = 4.5 Hz, H-3'); 7.38 (dd, 1H, *J* = 9.2 Hz and 2.5 Hz, H-7'); 5.77 (br ~d, *J*~9 Hz, 1H, H-9); 4.77 and 4.76 (2 × br s, 2 × 1H, H-2,5, η^5^-C_5_H_4_); 4.27 (br s, 2H, H-3,4, η^5^-C_5_H_4_); 4.03 (s, 5H, η^5^-C_5_H_5_); 3.95 (s, 3H, OCH_3_); 3.45 (br qa, *J* = 8.6 Hz, 1H, H-8); 3.23 (br ~t, *J* ~ 12 Hz, 1H, partly overlapped by the HDO signal of the solvent, H-6_α_); 3.12 (dd, *J* = 13.2 Hz and 9.5 Hz, 1H, H-2*_exo_*); 2.75 (ddd, *J* = 12.5 Hz, 11.4 Hz and 4.5 Hz, 1H, H-6_β_); 2.59 (ddd, *J *= 12.5 Hz, 11.2 Hz, 4.5 Hz, 1H, H-5_β_); 2.44 (br d, *J* = 13.2 Hz, 1H, H-2*_endo_*); 1.55 (br ~s, 1H, H-4); 1.53–1.48 (overlapping m’s, 2H, H-5_β_, H-7_β_); 1.43 (m, 1H, H-5_α_); 1.35 (m, 1H, H-3); 1.31 (m, 1H, CH_3_-CH_A_H_B_); 1.23 (m, 1H, CH_3_-CH_A_H_B_); 0.82 (t, 3H, *J* = 7.2 Hz, CH_3_); 0.68 (dd, *J* = 13.5 Hz and 8.1 Hz, 1H, H-7_α_); ^13^C-NMR (DMSO-d_6_): 169.2 (C=O); 158.1 (C-6'); 148.5 (C-2'); 146.2 (C-4'); 145.0 (C-8a'); 132.1 (C-8'); 129.3 (C-4a'); 122.0 (C-7'); 120.9 (C-3'); 103.9 (C-5'); 77.0 (C-1, η^5^-C_5_H_4_); 70.8 (two coalesced lines, C-3,4, η^5^-C_5_H_4_); 70.1 (η^5^-C_5_H_5_); 69.2 and 69.0 (C-2,5, η^5^-C_5_H_4_); 58.5 (C-8); 58.1 (C-2); 56.4 (OCH_3_); 50.0 (C-9); 41.9 (C-6); 37.8 (C-3); 29.1 (C-5); 27.9 (CH_3_-CH_2_); 27.0 (C-7); 25.5 (C-4); 12.9 (CH_3_-CH_2_); HRMS exact mass calculated for C_31_H_36_N_3_O_2_^56^Fe: 538.2157 [MH]^+^; found: 538.2162.

#### 3.2.2. N*-{(*S*)-[(2*S*,4*R*,8*R*)-8-Ethylquinuclidin-2-yl](6-methoxyquinolin-4-yl)methyl)}ferrocene-1,1'-bis-carboxamide* (**3**)

The amine (467 mg, 1.4 mmol), 1,1'-*bis-*fluorocarbonylferrocene (200 mg, 2.9 mmol; prepared from ferrocene dicarboxylic acid [39]) and DMAP (176 mg, 2.9 mmol) were dissolved in DCM (6 mL) and the solution was stirred under argon for 45 min. The residue obtained by the evaporation of the reaction mixture was purified by flash column chromatography on silica using DCM/MeOH (5:1) as eluent followed by recrystallization from Et_2_O to yield the product as brownish yellow powder (134 mg 21%). mp. 137.5–139.5 °C; [α]_D_^26^: −62.1° (EtOH *c *= 0.22 g/100 mL); IR (cm^−1^): 3248, 1645, 1623, 1608, 1533, 1509, 1229, 1175, 1030, 485; ^1^H-NMR (DMSO-d_6_): 8.71 (d, 1H, *J* = 4.5 Hz, H-2'); 8.02 (br s, 1H, NH); 7.93 (d, 1H, *J* = 9.2 Hz, H-8'); 7.85 (br s, 1H, H-5'); 7.63 (d, 1H, *J* = 4.5 Hz, H-3'); 7.40 (dd, 1H, *J* = 9.2 Hz and 2.5 Hz, H-7'); 5.72 (br ~d, *J*~8 Hz, 1H, H-9); 4.64 (br s, 2H, H-2,5, η^5^-C_5_H_4_); 4.53 and 4.50 (2 × br s, 2 × 1H, H-3,4, η^5^-C_5_H_4_); 3.94 (s, 3H, OCH_3_); 3.57 (br~t, *J* ~ 8 Hz,, 1H, H-8); 3.38 (m, 1H, H-6_α_); 3.16 (dd, *J* = 12.7 Hz and 7.3 Hz, 1H, H-2*_exo_*); 2.68 (ddd, *J *= 12.5 Hz, 11.2 Hz and 4.7 Hz, 1H, H-6_β_); 2.54 (br d, *J* = 12.7 Hz, 1H, H-2*_endo_*); 1.63–1.59 (overlapping m’s, 3H, H-4, H-5_β_, H-7_β_); 1.50-1.46 (overlapping m’s, 2H, H-3, H-5_α_); 1.42 (m, 1H, CH_3_-CH_A_H_B_); 1.35 (m, 1H, CH_3_-CH_A_H_B_); 0.83 (t, 3H, *J* = 7.2 Hz, CH_3_); 0.74 (dd, *J* = 13.2 Hz and 7.7 Hz, 1H, H-7_α_); ^13^C-NMR (DMSO-d_6_): 169.1 (C=O); 158.2 (C-6'); 148.6 (C-2'); 145.9 (C-4'); 145.1 (C-8a'); 132.2 (C-8'); 128.3 (C-4a'); 122.2 (C-7'); 121.0 (C-3'); 103.8 (C-5'); 78.0 (C-1, η^5^-C_5_H_4_); 72.4 (two coalesced lines, C-3,4, η^5^-C_5_H_4_); 70.7 (two coalesced lines, C-2,5, η^5^-C_5_H_4_); 58.7 (C-8); 58.0 (C-2); 56.5 (OCH_3_); 49.7 (C-9); 41.9 (C-6); 37.6 (C-3); 28.8 (C-5); 27.8 (CH_3_-CH_2_); 27.4 (C-7); 25.9 (C-4); 12.7 (CH_3_-CH_2_); HRMS exact mass calculated for C_52_H_61_N_6_O_4_^56^Fe: 889.4104 [MH]^+^; found: 889.4130.

#### 3.2.3. *1,1'-(Ferrocene-1,1'-diyl)-bis-{3-[(*S*)-((2*S*,4*R*,8*R*)-8-ethylquinuclidin-2-yl](6-methoxyquinolin-4-yl)methyl)}urea* (**4**)

The amine (7.58 g, 23 mmol) and 1,1'-diisocyanatoferrocene [40] (2.6 g, 9.7 mmol) were stirred in dry THF at RT under argon overnight. After the evaporation of the reaction mixture the crude product was dissolved in DCM/MeOH (10:1) and the solution was passed through Celite and concentrated in *vacuo*. The residue was column chromatographed on silica with DCM/MeOH (10:1) and crystallized by EtOH (15 mL) to obtain the pure product as yellow powder (358 mg, 2%). mp. 230 °C (dec.); [α]_D_^26^: −107.0° (EtOH *c *= 0.02 g/100 mL); IR (cm^−1^): 3277, 3244, ~3100–2100 (diffuse), 1678, 1622, 1584, 1510, 1247, 1083, 1029, 599, 486; ^1^H-NMR (CDCl_3_): 11.20 (br s, 1H, NH, bonded to η^5^-C_5_H_4_); 8.81 (s, 1H, NH, bonded to C-9); 8.76 (d, 1H, *J* = 4.5 Hz, H-2'); 8.03 (d, 1H, *J* = 9.2 Hz, H-8'); 7.98 (br s, 1H, H-5'); 7.46 (d, 1H, *J* = 4.5 Hz, H-3'); 7.40 (dd, 1H, *J* = 9.2 Hz and 2.5 Hz, H-7'); 5.93 (br ~d, *J* ~ 9 Hz, 1H, H-9); 5.02 and 4.13 (2 × br s, 2 × 1H, H-2,5, η^5^-C_5_H_4_); 4.51 (br qa, *J* = 8.8 Hz, 1H, H-8); 4.10 (s, 3H, OCH_3_); 4.04 (br ~t, *J*~13 Hz, 1H, H-6_α_); 3.83 and 3.56 (2 × br s, 2 × 1H, H-3,4, η^5^-C_5_H_4_); 3.74 (dd, *J *= 13.2 Hz and 9.5 Hz, 1H, H-2*_exo_*); 3.24–3.16 (overlapping m’s, 2H, H-2*_endo_*, H-6_α_); 2.18 (ddd, *J *= 12.5 Hz, 11.2 H and 4.5 Hz, 1H, H-6_β_); 2.03 (br s, H-4); 1.93–1.87 (m, 4H, H-3, H-5_α_, H-5_β_, H-7_β_); 1.49–1.45 (m, 2H, CH_3_-CH_2_); 0.94 (t, 3H, *J *= 7.2 Hz, CH_3_); 0.87 (dd, *J* = 13.2 and 8.8 Hz, 1H, H-7_α_); ^13^C-NMR (CDCl_3_): 159.0 (C-6'); 155.2 (C=O); 148.0 (C-2'); 145.4 (C-8a'); 142.6 (C-4'); 132.2 (C-8'); 128.5 (C-4a'); 122.8 (C-7'); 119.7 (C-3'); 102.5 (C-5'); 99.6 (C-1, η^5^-C_5_H_4_); 64.2 and 62.4 (C-3,4, η^5^-C_5_H_4_); 59.0 (two coalesced lines, C-2,5, η^5^-C_5_H_4_); 64.9 (C-8); 56.7 (C-2); 56.4 (OCH_3_); 50.0 (C-9); 41.2 (C-6); 35.3 (C-3); 26.9 (CH_3_-CH_2_); 25.34 (C-7); 25.30 (C-4); 25.2 (C-5); 11.9 (CH_3_-CH_2_); HRMS exact mass calculated for C_52_H_63_N_8_O_4_^56^Fe: 919.4322 [MH]^+^; found: 919.4352. 

#### 3.2.4. *1,1'-(Ferrocene-1,1'-dicarbonyl-diyl)-bis-{3-[(*S*)-((2*S*,4*R*,8*R*)-8-ethylquinuclidin-2-yl](6-methoxyquinolin-4-yl)methyl)}thiourea* (**5**)

1,1'-*bis-*Isothiocyanatocarbonylferrocene was prepared from 1,1'-*bis-*chlorocarbonylferrocene (2.18 g, 7 mmol) according to the procedure employing potassium isothiocyanate as reagent and acetone as solvent [41]. This reactive intermediate was used without purification after acetone was removed by distillation and the residue was dissolved in THF. The amine (5.39 g, 17 mmol) and 1,1'-*bis-*(isothiocyanatocarbonyl)ferrocene dissolved in 100 mL of dry THF were stirred overnight at RT under argon. The reaction mixture was concentrated in *vacuo*. The residue was subjected to column chromatography on silica using DCM/MeOH (15:1) as eluent. The partially purified product was dissolved in EtOH and slowly precipitated by water. The precipitate was filtered off then the chromatography and the recrystallization were repeated in order to get rid of the traces of isothiocyanate reagent to afford the pure product as brick-red powder (120 mg, 2%). mp. 167–169 °C; [α]_D_^26^: −128.6° (EtOH *c *= 0.22 g/100 mL); IR (cm^−1^): 3157, 1669, 1621, 1540, 1508, 1226, 1160, 1027, 592, 490; ^1^H-NMR (DMSO-d_6_): 11.62 (br s, 1H, NH inside the chelate); 10.70 (br s, 1H, NH outside the chelate); 8.74 (d, 1H, *J* = 4.5 Hz, H-2'); 7.98 (d, 1H, *J* = 9.2 Hz, H-8'); 7.83 (br s, 1H, H-5'); 7.58 (d, 1H, *J* = 4.5 Hz, H-3'); 7.44 (dd, 1H, *J* = 9.2 Hz and 2.5 Hz, H-7'); 5.97 (br ~d, *J*~10 Hz, 1H, H-9); 5.16 and 5.08 (2 × br s, 2H, H-2,5, η^5^-C_5_H_4_); 4.53 and 4.50 (2 × br s, 2 × 1H, H-3,4, η^5^-C_5_H_4_); 3.97 (s, 3H, OCH_3_); 3.50 (br t, *J *= 10.0 Hz, 1H, H-8); 3.23–3.16 (overlapping m’s, 2H, H-2*_exo_* and H-6_α_); 2.76 (ddd, *J* = 12.5 Hz, 11.2 H and 4.5 Hz, 1H, H-6_β_); 2.53 (dd, *J* = 13.8 Hz and 4.9 Hz, 1H, H-2*_endo_*); 1.68 (m, 1H, H-5_β_); 1.60 (br s, 1H, H-4); 1.50 (m, 1H, H-5_α_); 1.44 (m, 1H, H-3); 1.28 (br dd, *J* = 13.3 Hz and 10.0 Hz, 1H, H-7_β_); 1.27–1.22 (overlapping m’s, 2H, CH_3_-CH_2_); 0.95 (dd, *J* = 13.5 Hz and 8.2Hz, 1H, H-7_α_); 0.76 (t, 3H, *J *= 7.2 Hz, CH_3_); ^13^C-NMR (DMSO-d_6_): 180.4 (C=S); 171.6 (C=O); 158.2 (C-6'); 148.5 (C-2'); 144.5 (C-4'); 145.3 (C-8a'); 132.3 (C-8'); 128.7 (C-4a'); 122.0 (C-7'); 121.9 (C-3'); 104.0 (C-5'); 75.6 (C-1, η^5^-C_5_H_4_); 74.89 and 74.85 (C-3,4, η^5^-C_5_H_4_); 72.4 and 72.0 (C-2.5, η^5^-C_5_H_4_); 60.4 (C-8); 57.8 (C-2); 56.9 (OCH_3_); 56.5 (C-9); 42.2 (C-6); 37.6 (C-3); 28.7 (C-5); 27.5 (C-4); 26.1 (CH_3_-CH_2_); 25.7 (C-7); 12.5 (CH_3_-CH_2_); HRMS exact mass calculated for C_54_H_63_N_8_O_4_S_2_^56^Fe: 1007.3763 [MH]^+^; found: 1007.3776.

#### 3.2.5. *1-Benzoyl-3-[(*S*)-((2*S*,4*R*,8*R*)-8-ethylquinuclidin-2-yl](6-methoxyquinolin-4-yl)methyl)thiourea* (**6**)

The amine (3.00 g, 9.2 mmol) and benzoylisothiocyanate (1.51 g, 9.2 mmol) were dissolved in dry THF (100 mL). The reaction mixture was stirred overnight at RT and evaporated to dryness. The residue was purified by flash column chromatography on silica using DCM/MeOH (80:1) as eluent to obtain the product as glassy transparent substance (484 mg, 10%). mp. 92–94 °C; [α]_D_^26^: −217.3° (EtOH *c *= 0.31 g/100 mL); IR (cm^−1^): 3162, 1667, 1621, 1542, 1507, 1258, 1147, 1027; ^1^H-NMR (CDCl_3_): 11.50 (br s, 1H, NH inside the chelate); 11.20 (br s, 1H, NH outside the chelate); 8.64 (d, 1H, *J* = 4.5 Hz, H-2'); 7.88 (d, 1H, *J* = 9.2 Hz, H-8'); 7.84 (d, 2H, *J* = 7.3 Hz, H-2,6, Ph); 7.70 (br s, 1H, H-5'); 7.54 (t, 1H, *J* = 7.3 Hz, H-4, Ph); 7.51 (d, 1H, *J* = 4.5 Hz, H-3'); 7.35 (dd, 1H, *J* = 9.2 Hz and 2.5 Hz, H-7'); 5.78 (br ~d, *J*~8 Hz 1H, H-9); 3.86 (s, 3H, OCH_3_); 3.28 (br ~t, *J* ~ 8 Hz, 1H, H-8); 3.10–3.05 (overlapping m’s, 2H, H-2*_exo_* and H-6_α_); 2.58 (ddd, *J* = 12.5 Hz,11.2 Hz and 4.5 Hz, 1H, H-6_β_); 2.36 (dd, *J *= 13.8 Hz and 4.9 Hz, 1H, H-2*_endo_*); 1.54 (m, 1H, H-5_β_); 1.47 (m, 1H, H-4); 1.37 (m, 1H, H-5_α_); 1.28 (m, 1H, H-3); 1.15–1.05 (m, 3H, H-7_β_, CH_3_-CH_2_); 0.79 (dd, *J* = 13.5 Hz and 8.2Hz, 1H, H-7_α_); 0.67 (t, 3H, *J* = 7.2 Hz, CH_3_); ^13^C-NMR (CDCl_3_): 180.4 (C=S); 169.0 (C=O); 158. 0 (C-6'); 148.6 (C-2'); 145.5 (C-4'); 145.0 (C-8a'); 133.9 (C-4, Ph); 133.0 (C-1, Ph); 132.3 (C-8'); 129.5 (C-2,6, Ph); 129.3 (C-3,5, Ph); 128.7 (C-4a'); 122.0 (two coalesced lines, C-3', C-7'); 103.5 (C-5'); 60.0 (C-8); 57.7 (C-2); 56.4 (OCH_3_); 55.7 (C-9); 41.9 (C-6); 37.6 (C-3); 29.0 (C-5); 27.7 (C-4); 26.1 (CH_3_-CH_2_); 25.7 (C-7); 12.8 (CH_3_-CH_2_); HRMS exact mass calculated for C_28_H_33_N_4_O_2_S: 489.2324 [MH]^+^; found: 489.2323. 

#### 3.2.6. *1,3-Bis-{(*S*)-[(2*S*,4*R *8*R*)-8-ethylquinuclidin-2-yl](6-methoxyquinolin-4-yl)methyl)}thiourea* (**7**)

The amine (7.58 g, 23 mmol) and thiocarbonyldiimidazole (TCDI; 2.08 g, 12 mmol) were stirred in dry THF (150 mL) under argon. After TCDI was slowly dissolved the solution was evaporated and the residue was subjected to flash column chromatography on silica using DCM/MeOH (15:1) as eluent. The resulted oily substance was crystallized by water-ethanol and thoroughly washed with boiling water to obtain the product as white powder (171 mg, 2%). mp. 137–138 °C; [α]_D_^26^: −141.8° (EtOH *c *= 0.21 g/100 mL); IR (cm^−1^): 3265, 1622, 1541, 1509, 1257, 1082, 1031; ^1^H-NMR (DMSO-d_6_): 8.58 (d, 1H, *J* = 4.5 Hz, H-2'); 7.94 (br s, 1H, NH); 7.82 (d, 1H, *J* = 9.2 Hz, H-8'); 7.70 (br s, 1H, H-5'); 7.29 (dd, 1H, *J* = 9.2 Hz and 2.5 Hz, H-7'); 7.25 (d, 1H, *J* = 4.5 Hz, H-3'); 5.12 (br d, *J *= 10.0 Hz,1H, H-9); 3.81 (s, 3H, OCH_3_); 3.00 (dd, *J* = 12.9 Hz and 10.2 Hz, 1H, H-2*_exo_*); 2.95 (br t, *J* = 10.0 Hz, 1H, H-8); 2.85 (br ~t, *J* ~ 12 Hz, 1H, H-6_α_); 2.35 (br ~t, *J*~12 Hz 1H, H-6_β_); 2.23 (br d, *J* = 12.9 Hz, 1H, H-2*_endo_*); 1.38 (m, 2H, H-4, H-5_β_); 1.28 (m, 1H, H-3); 1.24 (m, 1H, H-5_α_); 1.10–1.05 (m, 2H, CH_3_-CH_2_); 0.99 (br dd, *J* = 13.3 Hz and 10.3Hz, 1H, H-7_β_); 0.69 (br d, *J *= 13.5 Hz, 1H, H-7_α_); 0.65 (t, 3H, *J* = 7.2 Hz, CH_3_); ^13^C-NMR (DMSO-d_6_): 183.0 (C=S); 157.9 (C-6'); 148.3 (C-2'); 147.0 (C-4'); 145.0 (C-8a'); 132.0 (C-8'); 128.9 (C-4a'); 121.9 (C-7'); 121.4 (C-3'); 104.1 (C-5'); 60.5 (C-8); 57.8 (C-2); 57.0 (C-9); 56.5 (OCH_3_); 41.6 (C-6); 37.7 (C-3); 29.0 (C-5); 27.6 (C-4); 26.2 (CH_3_-CH_2_); 25.9 (C-7); 12.6 (CH_3_-CH_2_); HRMS exact mass calculated for C_41_H_53_N_6_O_2_S: 693.3951 [MH]^+^; found: 693.3959. 

#### 3.2.7. N*-{(*S*)-[(2*S*,4*R*,8*R*)-8-Ethylquinuclidin-2-yl](6-methoxyquinolin-4-yl)methyl)}benzene-1,3,5-tris-carboxamide* (**8**)

The amine (3.60 g 11 mmol), pyridine (0.89 mL 11 mmol) and DMAP (224 mg 1.8 mmol) were dissolved in dry DCM (100 mL). During vigorous stirring 1,3,5-*tris-*chlorocarbonylbenzene (0.66 mL, 3.7 mmol) was added to the solution in one portion. After stirring for 24 h the solution was poured onto ice. DCM was distilled off at atmospheric pressure. The resulted precipitate was filtered off and dried to yield the product as white powder (3.60 g 87%). mp. 245 °C (dec.); [α]_D_^26^: −122.9° (EtOH *c *= 0.26 g/100 mL); IR (cm^−1^): 3305, 1658, 1621, 1508, 1229, 1029, 1029; ^1^H-NMR (CDCl_3_): 8.65 (d, 1H, *J* = 4.5 Hz, H-2'); 8.48 (s, 1H, H-2,4,6, Ph); 8.01 (d, 1H, *J* = 9.2 Hz, H-8'); 7.95 (br s, 1H, NH); 7.67 (br s, 1H, H-5'); 7.37 (dd, 1H, *J* = 9.2 Hz and 2.5 Hz, H-7'); 7.33 (d, 1H, *J* = 4.5 Hz, H-3'); 5.45 (br ~d, *J* ~ 10 Hz, 1H, H-9); 3.94 (s, 3H, OCH_3_); 3.19 (dd, *J* = 13.9 Hz and 9.6 Hz, 1H, H-2*_exo_*); 3.09–3.04 (overlapping m’s, 2H, H-6_α_ and H-8); 2.67 (m, 1H, H-6_β_); 2.38 (dd, *J* = 13.9 Hz and 5.0 Hz, 1H, H-2*_endo_*); 1.64 (br s,1H, H-4); 1.62 (m, 1H, H-5_β_); 1.53 (m, 1H, H-5_α_); 1.43 (m, 1H, H-3); 1.35 (ddd, *J* = 13.3 Hz, 10.3 Hz and 5.0 Hz, 1H, H-7_β_); 1.25 and 1.19 (2 × m, 2 × 1H, CH_3_-CH_2_); 0.96 (dd, *J* = 13.3 Hz and 6.4 Hz, 1H, H-7_α_); 0.78 (t, 3H, *J *= 7.2 Hz, CH_3_); ^13^C-NMR (CDCl_3_): 165.8 (C=O); 158.3 (C-6'); 148.0 (C-2'); 145.8 (C-8'); 145.6 (C-4a); 134.7 (C-1,3,5 Ph); 132.3 (C-8'); 129.4 (C-2,4,6, Ph); 128.6 (C-4a'); 122.0 (C-7'); 119.1 (C-3'); 102.1 (C-5'); 60.7 (C-8); 56.7 (C-2); 56.0 (OCH_3_); 51.7 (C-9); 41.4 (C-6); 37.5 (C-3); 29.0 (C-5); 27.7 (CH_3_-CH_2_); 26.1 (C-4); 25.5 (C-7); 12.3 (CH_3_-CH_2_); HRMS exact mass calculated for C_69_H_82_N_9_O_6_: 1132.6421 [MH]^+^; found: 1132.6388.

### 3.3. *In Vitro* Cytostatic and Cytotoxic Activity of the Compounds

The cells were grown to confluency and were plated into 96-well plate with initial cell number of 5.0–7.5 × 10^3^ per well. After 24 h incubation at 37 °C, cells were treated with the compounds in 200 μL final volume containing 1.0 v/v% DMSO. Cells were incubated with the compounds at 10^−4^–10^2^ μM concentration range for overnight. Control cells were treated with serum free medium (RPMI-1640 or DMEM) only or with DMSO (c = 1.0 v/v%) at 37 °C for overnight. After incubation the cells were washed twice with serum free (RPMI-1640 or DMEM) medium. To determine the *in vitro* cytostatic effect, cells were cultured for a further 72 h in serum containing medium. To measure the *in vitro* cytotoxicity of the compounds, MTT-assay was carried out immediately after the overnight treatment: The cell viability was determined by the following method using 3-(4,5-dimethylthiazol-2-yl)-2,5-diphenyltetrazolium bromide [42,43]. The solution of MTT (45 μL, 2 mg/mL) was added to each well which was reduced by the respiratory chain [42,43] and other electron transport systems [44] to form precipitated violet formazan crystals within the cell [45]. The amount of these crystals can be determined by spectrophotometry serving as an estimate for the number of mitochondria and hence the number of living cells in the well [46]. After 4 h of incubation the cells were centrifuged for 5 min (900 g) and supernatant was removed. The obtained formazan crystals were dissolved in 50 μL of DMSO and the optical density (OD) of the samples was measured at λ = 540 and 620 nm, respectively, employing ELISA Reader instrument (iEMS Reader, Labsystems, Finland). OD_620_ values were subtracted from OD_540_ values and the percent of cytostasis or cytotoxicity was calculated using equation “Cytostatic effect/Cytotoxicity (%) = [1 − (ODtreated/ODcontrol)] × 100” (where OD_treated_ and OD_control_ correspond to the optical densities of the treated and the control cells, respectively). In each case two independent experiments were carried out with 4–8 parallel measurements. The 50% inhibitory concentration (IC_50_) values were determined from the dose-response curves. The curves were defined using Microcal^TM^ Origin1 (version 7.5) software: cytostasis (%) or cytotoxicty (%) was plotted as a function of concentration, fitted to a sigmoidal curve and, based on this curve, the half maximal inhibitory concentration (IC_50_) value was determined representing the concentration of a compound required for 50% inhibition *in vitro* and expressed in micromoles.

## 4. Conclusions

Among the novel compounds reported in this contribution the *bis*- and *tris*-quinine derivatives exerted a dose-dependent *in vitro* antitumor activity at micromolar concentrations on the investigated tumor cell cultures. The ferrocene-based *bis*-amide **3** of pronounced activity can be considered as a promising lead structure for the development of a novel class of therapeutical agents. The highly promising results also obtained for a metallocene model containing two ureido functional groups (compound **4**) suggest that 1,1'-disubstituted ferrocene unit with easily rotating Cp-rings seems to be highly beneficial to the desired activity allowing the molecule to adopt a conformation in which the two cooperating groups are situated in optimal distance from each other. On the other hand, the presence of rigid acylthiourea moiety stabilized by chelate structure dramatically decreases the activity on the investigated cell lines.

## References

[1] Ding Y., Bao Y., An L. (2005). Progress in antitumor agents, vinblastine analogues. Zhongguo Yiyao Gongye Zazhi.

[2] Gao H. (2008). Research status of antitumor drug camptothecin and its derivatives. Hebei Yiyao.

[3] Prudhomme M. (2005). Staurosporines and structurally related indolocarbazoles as antitumor agents. Anticancer Agents Nat. Prod..

[4] Ohashi M., Oki T. (1996). Ellipticine and related anticancer agents. Expert Opin. Ther. Pat..

[5] Kaur K., Jain M., Reddy R.P., Jain R. (2010). Quinolines and structurally related heterocycles as antimalarials. Eur. J. Med. Chem..

[6] Wolf R., Baroni A., Greco R., Donnarumma G., Ruocco E., Tufano M.A., Ruocco V. (2002). Quinine sulfate and bacterial invasion. Ann. Clin. Microbiol. Antimicrob..

[7] Kelsey F.E., Brunschwig A. (1947). Concentration of quinine in gastrointestinal cancers; preliminary report. Cancer Res..

[8] Kim J., Lee K., Jung W., Lee O., Kim T., Kim H., Lee J., Passaro D.J. (2005). Validity of serum pepsinogen levels and quininium resin test combined for gastric cancer screening. Cancer Detect. Prev..

[9] Lehnert M., Dalton W.S., Roe D., Emerson S., Salmon S.E. (1991). Synergistic inhibition by verapamil and quinine of P-glycoprotein-mediated multidrug resistance in a human myeloma cell line model. Blood.

[10] Taylor C.W., Dalton W.S., Mosley K., Dorr R.T., Salmon S.E. (1997). Combination chemotherapy with cyclophosphamide, vincristine, adriamycin, and dexamethasone (CVAD) plus oral quinine and verapamil in patients with advanced breast cancer. Breast Cancer Res. Treat..

[11] Genne P., Dimanche-Boitrel M.T., Mauvernay R.Y., Gutierrez G., Duchamp O., Petit J.M., Martin F., Chauffert B. (1992). Cinchonine, a potent efflux inhibitor to circumvent anthracycline resistance *in vivo*. Cancer Res..

[12] Baraniak D., Kacprzak K., Celewicz L. (2011). Synthesis of 3′-azido-3′-deoxythymidine (AZT) cinchona alkaloid conjugates via click chemistry: Toward novel fluorescent markers and cytostatic agents. Bioorg. Med. Chem. Lett..

[13] Sohue N. (1941). Quinine derivatives and the transplantable tumor. III. The effect of quinine derivatives upon the growth rate of Fujinawa's rat sarcoma in the tissue culture. Folia Pharm. Jpn..

[14] Sakai S., Minoda K., Saito G., Akagi S., Ueno A., Fukuoka F. (1955). The anticancer action of quinoline derivatives. Gann.

[15] Fiorina V.J., Dubois R.J., Brynes S. (1978). Ferrocenyl polyamines as agents for the chemoimmunotherapy of cancer. J. Med. Chem..

[16] Koepf-Maier P., Koepf H., Neuse E.W. (1984). Ferrocenium salts—The first antitumor iron compounds. Angew. Chem. Int. Ed..

[17] Neuse E.W., Kanzawa F. (1990). Evaluation of the activity of some water-soluble ferrocene and ferricenium compounds against carcinoma of the lung by the human tumor clonogenic assay. Appl. Org.-Met. Chem..

[18] Snegur L.V., Nekrasov S., Gumenyuk V.V., Zhilina Z.V., Morozova N.B., Skviridova I.K., Rodina I.A., Sergeeva N.S., Shchitkov K.G. (1998). Ferrocenylalkylazoles, a new class of low-toxicity compounds with antitumor activity. Rossiiskii Khim. Zhurn..

[19] Osella D., Ferrali M., Zanello P., Laschi F., Fontani M., Nervi C., Cavigiolio G. (2000). On the mechanism of the antitumor activity of ferrocenium derivative. Inorg. Chim. Acta.

[20] Gormen M., Pigeon P., Top S., Vessieres A., Plamont M.A., Hillard E.A., Jaouen G. (2010). Facile synthesis and strong antiproliferative activity of disubstituted diphenylmethylidenyl-[3]ferrocenophanes on breast and prostate cancer cell lines. Med. Chem. Commun..

[21] Monserrat J.P., Chabot G.G., Hamon L., Quentin L., Scherman D., Jaouen G., Hillard E.A. (2010). Synthesis of cytotoxic ferrocenyl flavones via a ferricenium-mediated 1,6-oxidative cyclization. Chem. Commun..

[22] Hillard E.A., Vessieres A., Thouin L., Jaouen G., Amatore C. (2006). Ferrocene-mediated proton-coupled electron transfer in a series of ferrocifen -type breast-cancer drug candidates. Angew. Chem. Int. Ed..

[23] Li H., Lv P., Yan T., Zhu H. (2009). Urea derivatives as anticancer agents. Anticancer Agents Med. Chem..

[24] Jordan A.M., Khan T.H., Malkin H., Osborn H.M.I. (2002). Synthesis and analysis of urea and carbamate prodrugs as candidates for melanocyte-directed enzyme prodrug therapy (MDEPT). Bioorg. Med. Chem..

[25] Ma Z., Saluta G., Kucera G.L., Bierbach U. (2008). Effect of linkage geometry on biological activity in thiourea and guanidine-substituted acridines and platinum-acridines. Bioorg. Med. Chem. Lett..

[26] Cesarini S., Spallarossa A., Ranise A., Schenone S., Rosano C., La Colla P., Sanna G., Busonera B., Loddo R. (2009). *N*-Acylated and *N,N'*-diacylated imidazolidine-2-thione derivatives and *N,N'*-diacylated tetrahydropyrimidine-2(1*H*)-thione analogues: Synthesis and antiproliferative activity. Eur. J. Med. Chem..

[27] Rao X., Wu Y., Song Z., Shang S., Wang Z. (2011). Synthesis and antitumor activities of unsymmetrically disubstituted acylthioureas fused with hydrophenanthrene structure. Med. Chem. Res..

[28] Suda Y., Egami K., Fujita H. (2009). Preparation of acylthiourea compounds as c-Met kinase inhibitors. PCT Int. Appl..

[29] Ruat M., Faure H., Traiffort E., Schoenfelder A., Mann A., Taddei M., Solinas A., Manetti F. (2009). Preparation of aromatic *N*- acylthiourea and *N*-acylurea as inhibitors of the Hedgehog protein signalling pathway for the treatment of cancer, neurodegenerative diseases and diabetes. Fr. Demande.

[30] Garcia-Martin F., Cruz L.J., Rodriguez-Mias R.A., Giralt E., Albericio F. (2008). Design and synthesis of FAJANU: A de Novo C2 symmetric cyclopeptide family. J. Med. Chem..

[31] Manna C.M., Tshuva E.Y. (2010). arkedly different cytotoxicity of the two enantiomers of C2-symmetrical Ti(IV) phenolato complexes; mechanistic implications. Dalton T..

[32] Rabouin D., Perron V., N'Zemba B.C., Gaudreault R., Berube G. (2003). A facile synthesis of C2-symmetric 17b-estradiol dimers. Bioorg. Med. Chem. Lett..

[33] Raynes K., Galatis D., Cowman A.F., Tilley L., Deady L.W. (1995). Synthesis and activity of some antimalarial bisquinolines. J. Med. Chem..

[34] Ayad F., Tilley L., Deady L.W. (2001). Synthesis, antimalarial activity and inhibition of haem detoxification of novel bisquinolines. Bioorg. Med. Chem. Lett..

[35] Cowman A.F., Deady L.W., Deharo E., Desneves J., Tilley L. (1997). Synthesis and activity of some antimalarial bisquinolinemethanols. Aust. J. Chem..

[36] Brunner H., Buegler J. (1997). Enantioselective catalysis. 106. 9-Amino-(9-deoxy)cinchona alkaloids and their derivatives. B. Soc. Chim. Belg..

[37] Vakulya B., Varga S., Csámpai A., Soós T. (2005). Highly enantioselective conjugate addition of nitromethane to chalcones using bifunctional cinchona organocatalysts. Org. Lett..

[38] Frisch M.J., Trucks G.W., Schlegel H.B., Scuseria G.E., Robb M.A., Cheeseman J.R., Montgomery J.A., Vreven T., Kudin K.N., Burant J.C. (2003). GAUSSIAN 03, Rev. A. 1.

[39] Galow T.H., Rodrigo J., Cleary K., Cooke G., Rotello V.M. (1999). Fluorocarbonylferrocene. A versatile intermediate for ferrocene esters and amides. J. Org. Chem..

[40] Van Leusen D., Hessen B. (2001). 1,1'-Diisocyanoferrocene and a convenient synthesis of ferrocenylamine. Organometallics.

[41] Yuan Y., Ye S., Zhang L., Wang B., Xu Y., Wang J., Wang H. (1997). Studies on intramolecular hydrogen bonding of 1,1'-bis[*N*-formyl-*N'*-*p*-chlorophenylthiourea]ferrocene. Inorg. Chim. Acta.

[42] Slater T.F., Sawyer B., Sträuli U. (1963). Studies on succinate-tetrazolium reductase systems: III. Points of coupling of four different tetrazolium salts III. Points of coupling of four different tetrazolium salts. Biochim. Biophys. Acta.

[43] Mosmann T. (1983). Rapid colorimetric assay for cellular growth and survival: Application to proliferation and cytotoxicity assays. J. Immunol. Methods.

[44] Liu Y.B., Peterson D.A., Kimura H., Schubert D. (1997). Mechanism of cellular 3-(4,5-dimethylthiazol-2-yl)-2,5-diphenyltetrazolium bromide (MTT) reduction. J. Neurochem..

[45] Altman F.P. (1976). Tetrazolium salts and formazans. Prog. Histochem. Cytochem..

[46] Denizot F., Lang R. (1986). Rapid colorimetric assay for cell growth and survival: Modifications to the tetrazolium dye procedure giving improved sensitivity and reliability. J. Immunol. Methods.

[47] Datki Z., Juhász A., Gálfi M., Soós K., Papp R., Zádori D., Penke B. (2003). Method for measuring neurotoxicity of aggregating polypeptides with the MTT assay on differentiated neuroblastoma cells. Brain Res. Bull..

[48] Datki Z., Papp R., Zádori D., Soós K., Fülöp L., Juhász A., Laskay G., Hetényi C., Mihalik E., Zarándi M., Penke B. (2004). *In vitro* model of neurotoxicity of Aβ 1-42 and neuroprotection by a pentapeptide: irreversible events during the first hour. Neurobiol. Dis..

[49] Biedler J.L., Roffler-Tarlov S., Schachner M., Freedman L.S. (1978). Multiple Neurotransmitter Synthesis by Human Neuroblastoma Cell Lines and Clones. Cancer Res..

[50] Biedler J.L., Helson L., Spengler B.A. (1973). Morphology and growth, tumorigenicity, and cytogenetics of human neuroblastoma cells in continuous culture. Cancer Res..

